# Co-Fermentation by *Lactobacillus brevis* B7 Improves the Antioxidant and Immunomodulatory Activities of Hydroponic Ginseng-Fortified Yogurt

**DOI:** 10.3390/antiox10091447

**Published:** 2021-09-13

**Authors:** Myung-Wook Song, Ji-Young Park, Hyun-Sook Lee, Kee-Tae Kim, Hyun-Dong Paik

**Affiliations:** 1 Department of Food Science and Biotechnology of Animal Resources, Konkuk University, Seoul 05029, Korea; carebie251@hanmail.net (M.-W.S.); parkgos2@naver.com (J.-Y.P.); richard44@hanmail.net (K.-T.K.); 2 Department of Food Service Management and Nutrition, Sangmyung University, Seoul 03016, Korea; leehs9292@smu.ac.kr

**Keywords:** co-fermentation, hydroponic ginseng, probiotics, yogurt, antioxidant activity, immunomodulatory activity

## Abstract

The development of convenient and accessible health-functional foods has become an area of increased interest in recent years. Probiotics, ginseng, and yogurts have been recognized as representative nutraceutical products. To improve the functionality of yogurts, co-fermentation was performed during yogurt preparation. Four kinds of yogurt were prepared using a combination of probiotic *Lactobacillus brevis* B7 and hydroponic ginseng based on plain yogurt. The fundamental characteristics of yogurts, including pH, titratable acidity, microbial counts, color, and physicochemical properties, were determined. To assess functionality, four different antioxidant assays and real-time PCR analysis using RAW 264.7 cells were performed. Finally, sensory evaluation was conducted to evaluate customer preference. Hydroponic ginseng supplementation influenced pH, solid content, lightness, and yellowness. However, probiotic supplementation did not affect most factors except pH. In functionality analysis, the yogurt co-fermented with probiotics and ginseng showed the highest antioxidant activity and gene expression levels of the immune-related factors TNF-α and iNOS in RAW 264.7 cells. Although ginseng supplementation received poor acceptance because of its color and flavor, these attempts were considered beneficial despite the risk. Overall, co-fermentation within a short yogurt preparation time presented the potential for improvement of functionality. These findings suggest a range of feasibility for the development of attractive nutraceutical products.

## 1. Introduction

Herbs, spices, and fruits derived from different parts of plants have long been used as food additives to enhance the organoleptic properties of foods, such as flavor, taste, and aroma. Such derivatives can function as food preservatives to improve shelf-life by suppressing or eradicating food-borne pathogens [1]. Furthermore, the functional properties of herbs and spices, such as antioxidant, anti-inflammatory, immunomodulatory, and anti-mutagenic activities, have also received attention. Several phenolic compounds from plants are excellent alternatives to artificial antimicrobial and antioxidant ingredients. Catechins, ellagic acid, coumaric acid, ferulic acid, and oleuropein are representative examples of functional phenolic compounds [2]. Herbs and spices are applied in different forms, such as extract, powder, essential oil, and fresh raw preparations.

Ginseng (*Panax ginseng* Meyer) is a powerful functional plant that is widely consumed for medicinal purposes. Ginseng has anti-aging, antioxidant, anti-inflammatory, anti-carcinogenic, and anti-obesity activities and consists of various polysaccharides, flavonoids, volatile oils, polyynes, and, particularly, ginsenosides with abundant pharmaceutical activity [3]. Hydroponically cultivated ginseng (without soil) has diverse advantages such as better productivity, low cost, uniform maintenance of environmental factors, and high functionality compared to traditional soil-cultivated ginseng. Furthermore, abundant contents of ginsenosides Rd, Re, and Rg_1_ are found in hydroponically cultivated ginseng [4]. Another merit of hydroponic ginseng is that this mode of cultivation enables the utilization of all parts of ginseng, including berries, leaves, and stems, which are generally discarded at harvest because of accumulated pesticides, even though they contain high contents of ginsenosides.

Probiotics are edible microorganisms with functional characteristics within the intestinal tract. These health-enhancing microbes can regulate intestinal infection, alleviate lactose intolerance, decrease serum cholesterol levels, and inhibit carcinogenesis [5]. Recently, probiotics have been regarded as a promising approach in research, pharmaceutical, and nutraceutical fields because of increased interest in the microbiome. The microbiome involves the interaction of microorganisms with the human body and the characteristics of the complex microbial community. To put the knowledge of the microbiome into action for human health, the establishment of certified delivery systems for probiotics from the manufacturing stages to settlement in the intestinal tract is essential. In this study, the *Lactobacillus brevis* B7 strain, isolated from kimchi, was used to enhance the functional properties of yogurt. This strain exhibits probiotic and health-functional activities and also presents β-glucosidase activity [6]. Therefore, through the co-fermentation with hydroponic ginseng and probiotics, improved functional properties are anticipated.

Currently, dairy products are considered representative food deliverers that have been effectively utilized to transport phytochemicals or probiotics. In particular, the application of probiotic bacteria to dairy products represents the potential for the bioconversion of food components through fermentation. These microorganisms metabolize the components of raw materials in foods using their innate enzymatic activity. During these interactions, lactose in dairy products is degraded by β-galactosidase; these reactions could be beneficial for individuals with lactose digestion issues. Furthermore, several proteins are also hydrolyzed, and bioactive peptides or polyamines, which have anti-hypertensive, antioxidant, immunomodulatory, and anti-thrombotic effects, are produced [7,8]. Phenolic compounds are also metabolized by lactic acid bacteria through various enzymes such as phenolic acid reductase, esterase, decarboxylase, and glycosyl hydrolase [9]. In addition, several unfavorable or toxic compounds, such as phytic acid, are discarded by providing optimal conditions for phytase activity during fermentation [10]. The combination of several functional agents and dairy products makes conventional dairy foods more valuable, owing to their enhanced nutraceutical properties, and increases competitiveness in the market [11].

Yogurt is a representative example of a food carrier because of its accessibility and potential for combination with various fruits and vegetables [8]. In particular, fermented dairy foods can serve as vehicles for food ingredients as well as probiotics. The food matrix of fermented dairy foods has the potential to protect these additives from acidic environments within the digestive tract by buffering the capacity of milk protein [12]. Fat content also has an effect on the stable transport of phytochemicals and probiotics through the prevention of direct exposure to bile salt or gastric acid. In a previous study, a greater number of bacterial cells survived in probiotic ice cream with high fat content than in that without fat content [13]. Since fermented dairy foods already possess functional properties, enhancement of functionality through supplementation with other food ingredients is anticipated to succeed.

Although supplementation with probiotics, ginseng, or fermented ginseng in dairy foods has already been investigated in a number of previous studies, the application of unfermented ginseng and probiotics together with yogurt is infrequent. Therefore, in this study, both a probiotic strain and hydroponic ginseng were supplemented in yogurts to improve their functionality and investigate their potential as a consumer product.

## 2. Materials and Methods

### 2.1. Materials for Yogurt Preparation

Whole cow milk, skim milk powder (Seoul Milk Co., Seoul, Korea), and commercial starter culture powder (ABT-B: *Lactobacillus acidophilus*, *Bifidobacterium longum*, and *Streptococcus thermophilus*, Samilk Dairy & Food Co., Seoul, Korea) were purchased to prepare yogurt. Hydroponic ginseng (Chungjung-Saessacksam Co., Gwangju, Korea) was purchased from a local market, washed with tap water, diced into small cubes, and dried at 60 °C. The prepared ginseng samples were pulverized, and 25 mg of ginseng powder was extracted with 500 mL of 70% ethyl alcohol for 6 h at 70 °C using the reflux method; these processes were repeated thrice using the remaining ginseng powder residue. The collected extracts were filtered using Whatman No. 2 filter paper and concentrated using a rotary evaporator (N-1000V, EYELA, Tokyo, Japan) at 60 °C. Concentrated dark-brown ginseng syrups were stored at −20 °C until use. All other chemicals were obtained from Sigma-Aldrich (St. Louis, MO, USA).

### 2.2. HPLC Analysis of Hydroponic Ginseng Extract

The ginsenoside composition of ginseng extract was assessed through the HPLC analysis using ten kinds of standard ginsenosides (Rb_1_, Rb_2_, Rb_3_, Rc, Rd, Re, Rf, Rg_3_, F_2_, and CK) material obtained from Biopurify Phytochemicals Ltd. (Chengdu, China). To prepare the samples, ten-fold diluted ginseng extract through HPLC-grade methyl alcohol was filtered by a Sep-Pak^®®^ Plus C18 cartridge filter (Waters, Milfore, MA, USA), and the adsorbed fraction of the samples was eluted using 30% methyl alcohol. The collected eluent was filtered again using a 0.20 μm syringe filter (Advantec, Tokyo, Japan).

Ginsenoside composition was analyzed using the Water 600 HPLC system (Water, Milford, MA, USA) with a reverse-phase column (Eclipse XDB-C18, 4.6 × 150 mm, 5 μm) (Agilent Technologies, Santa Clara, CA, USA). Two kinds of solvent, such as water (A) and acetonitrile (B), were used with the following gradients: 0 min (A 72%), 0–7 min (A 60%), 7–15 min (A 50%), 15–20 min (A 25%), and 20–25 min (A 72%). The flow speed was 1 mL/min, and the injection volume was 10 μL. The results were detected through a UV detector at 203 nm. The concentration of ginsenosides within the ginseng extract was calculated by the ratio of peak area between standard materials and the sample. The calibration curves of each standard were as follows: Re (Y = 0.0365X − 0.1804, R^2^ = 0.9989); Rf (Y = 0.0229X − 0.1776, R^2^ = 0.9997); Rb_1_ (Y = 0.0303X + 0.058, R^2^ = 0.9998); Rc (Y = 0.0276X − 0.002, R^2^ = 0.9996); Rb_2_ (Y = 0.0321X − 0.034, R^2^ = 0.9999); Rb_3_ (Y = 0.0308X + 0.056, R^2^ = 0.9998); Rd (Y = 0.0358X − 0.349, R^2^ = 0.9986); F_2_ (Y = 0.0359X − 0.0596, R^2^ = 0.9997); Rg_3_ (Y = 0.0389X − 0.038, R^2^ = 0.9997); CK (Y = 0.0507X − 0.04, R^2^ = 0.9999). X represents the concentration (μg/mL), and Y represents the peak area.

### 2.3. Preparation of Yogurt and Its Water-Soluble Extract

Four different types of yogurts—control yogurt (C), *Lactobacillus brevis* B7-supplemented yogurt (LB), hydroponic ginseng extract-supplemented yogurt (HG), and *L. brevis* B7 and hydroponic ginseng extract-supplemented yogurt (LHG)—were used in this study. The probiotic strain *L. brevis* B7 isolated from kimchi was pre-cultured at 37 °C for 15 h in MRS broth [6]. Before the preparation of yogurt, skim milk powder was added to milk to fit a concentration of 11% solid matter, and hydroponic ginseng extract was added into milk corresponding to HG and LHG with 0.5% (*w/v*) concentration according to the solid matter of ginseng extract (159.51 mg/mL) estimated by the hot-air drying method. The prepared milk samples were sterilized at 90 °C for 10 min. After sterilization, each milk sample was cooled to room temperature (25 °C), and then 0.1% (*w/v*) of the starter culture was inoculated. For probiotic yogurts, 1% (*v/v*) of cultured *L. brevis* B7 suspension was added to LB and LHG. Yogurts were fermented at 40 °C without agitation until the pH decreased to 4.5 ± 0.05. The prepared yogurts were stored in a refrigerator at 4 °C for at least 1 day for ripening.

The water-soluble extracts (supernatants) of yogurts were collected for further analysis [14]. Each yogurt (10 g) was diluted with sterilized distilled water (2.5 mL) and homogenized by agitation. The pH of diluted yogurts was adjusted to 7.0 ± 0.05 by adding NaOH (0.1 N), and the yogurts were centrifuged at 14,000× *g* for 15 min. The collected supernatants were filtered using a 0.45 μm syringe filter. The acquired clear water-soluble extracts of yogurts were stored at −20 °C until further analysis.

### 2.4. Physicochemical and Microbial Properties of Yogurts

The physicochemical properties of yogurts, including fat, protein, lactose, and total solids content, were determined with MilkoScan™ Minor (Type 78110, FOSS, Hillerød, Denmark) according to the manufacturer’s instructions. pH, titratable acidity, color, and ash content were measured according to the instructions provided by the Association of Official Analytical Chemists (AOAC, 2000); the pH was assessed using a pH meter (pH 7110, WTW, Weilheim, Germany), and color characteristics were assessed using a colorimeter (CR-400, Konica Minolta, Tokyo, Japan). Changes in the growth patterns of total bacteria were estimated by plate counting on bromocresol purple (BCP) medium (MB cell, Seoul, Korea) every hour during yogurt fermentation.

### 2.5. Antioxidant Activity of Yogurt

Four kinds of antioxidant assays were performed to estimate the antioxidant activities of the yogurts, as described by Lee et al. [15]. The antioxidant activities of the yogurts were measured using the yogurt supernatants mentioned above.

#### 2.5.1. DPPH Radical-Scavenging Activity

A DPPH (2,2-diphenyl-1-picryl-hydrazyl) solution was prepared by dissolving DPPH powder in 99.9% ethyl alcohol to adjust to 0.1 mM concentration, with an optical density of 1.2 ± 0.05 at 517 nm. Each yogurt sample was mixed with the DPPH solution at a 1:9 ratio and allowed to react for 30 min under dark conditions. Absorbance was measured at 517 nm.

#### 2.5.2. ABTS Radical-Scavenging Activity

ABTS (2-azinobis-(3-ethylbenzothiazoline-6-sulfonic acid) diammonium salt) stock solution was prepared by mixing 14 mM ABTS and 5 mM potassium persulfate suspended in 0.1 M potassium phosphate buffer (pH 7.4) in a 1:1 ratio. The mixture was allowed to react for 16 h at room temperature, after which it was diluted to an optical density of 0.7 ± 0.05 at 734 nm. Each yogurt sample was mixed with the ABTS solution at a 1:19 ratio and incubated for 15 min in the dark. Absorbance was measured at 734 nm.

DPPH and ABTS radical scavenging activities were calculated with the following equation:

(1) $$\mathrm{Radical}\;\mathrm{scavenging}\;\mathrm{activity}\;\left(\%\right)=\left(1-\frac{A_{\mathrm{sample}}}{A_{\mathrm{control}}}\right)\times 100$$

#### 2.5.3. Reducing-Power Activity

Fifty microliters of yogurt samples were allowed to react with 250 μL of 0.2 M sodium phosphate buffer (pH 6.6) and 1% potassium ferricyanide (pH 6.6) at 50 °C in a water bath for 20 min. After heat treatment, 250 μL of 10% trichloroacetic acid was added. Four hundred microliters of distilled water and 100 μL of 0.1% ferric chloride were added to 500 μL of the mixed solution and allowed to react for 30 min in the dark. The results were measured at 700 nm and calculated using the L-cysteine standard curve.

#### 2.5.4. FRAP Activity

FRAP solution was prepared by blending 300 mM acetate buffer (pH 3.6), 10 mM TPTZ (2,4,6-tri[2 -pyridyl]-s-triazine) suspended in 40 mM HCl, and 20 mM ferric chloride in a 10:1:1 ratio, and the mixture was allowed to react for 15 min at 50 °C in a water bath. Fifty microliters of each yogurt sample were allowed to react with 950 μL of FRAP solution for 30 min under dark conditions. The results were measured at 593 nm, then calculated using a ferrous sulfate standard curve.

### 2.6. Estimation of Cytotoxicity and Nitric Oxide Productivity

To estimate the cytotoxicity and immunomodulatory activities of yogurt samples, MTT and NO assays were performed in RAW 264.7 murine macrophage cells. RAW 264.7 cells were obtained from the Korean Cell Line Bank (KCLB, Seoul, Korea) and cultured in Dulbecco’s modified Eagle’s medium (DMEM), including 10% fetal bovine serum (FBS) and 1% penicillin/streptomycin (P/S) at 37 °C under a 5% CO_2_ environment [6].

#### 2.6.1. MTT Assay for Estimation of Cytotoxicity

One hundred microliters of pre-cultured RAW 264.7 cells were diluted and seeded into 96-well plates at a concentration of 2 × 10^5^ cells/well and incubated for 2 h. Then, equal volumes (50 μL) of DMEM and 2-fold serially diluted yogurt samples were mixed and incubated for 24 h. Lipopolysaccharide (LPS) treatment (1 ng/mL) was used as the control treatment for immunomodulatory activity. Subsequently, the cultured media were discarded, and cells were washed with PBS buffer. One hundred microliters of MTT solution (0.5 mg/mL) were added to the cells and incubated for 1 h. After incubation, the MTT solution was discarded, and the produced formazan crystals were dissolved in 100 μL of dimethyl sulfoxide. The absorbance was measured at 570 nm and calculated using the following equation:

(2) $$\mathrm{Cell}\;\mathrm{viability}\;\left(\%\right)=\frac{A_{\mathrm{with}\;\mathrm{sample}}}{A_{\mathrm{without}\;\mathrm{sample}}}\times 100$$

#### 2.6.2. NO Assay for Estimation of Immunomodulatory Activity

Cell preparation and sample treatment were performed identically using an MTT assay. After sample treatment and 24 h of incubation, 100 μL of supernatant from cell-cultured media was transferred into another well plate and allowed to react with an equal volume of Griess reagent to assess the amount of NO production. The absorbance was measured at 540 nm and calculated using a sodium nitrite standard curve.

### 2.7. Immunomodulatory Activity of Yogurts

To investigate the immunomodulatory activity of the yogurts, real-time PCR analysis was performed as described by Song et al. [6]. Two milliliters of cultured RAW 264.7 cells (1 × 10^6^ cells/mL) were seeded and incubated for 24 h. Then, 1 mL of sample/LPS and DMEM media were added to the cells and incubated for 24 h. RNA was extracted from RAW 264.7 cells using the RNeasy Mini Kit (Qiagen, Hilden, Germany) according to the manufacturer’s instructions. Subsequently, the extracted RNA was quantified, and cDNA synthesis was performed using a cDNA synthesis kit (Thermo-Fisher Scientific, Waltham, MA, USA).

To assess immunomodulatory activity, immune-related factors such as tumor necrosis factor (TNF)-α, inducible nitric oxide synthase (iNOS), interleukin (IL)-1β, and IL-6 were measured, and β-actin was used as a housekeeping gene and control. The degree of gene expression was assessed via quantitative reverse transcription PCR, using a real-time PCR system (PikoReal-96, Thermo-Fisher Scientific, Waltham, MA, USA), by detecting the fluorescence signal from SYBR green. The relative expression levels were calculated using the 2^−∆∆Cq^ (Cq, the number of cycles when the fluorescence signal was detected) method and normalized to β-actin expression. Forward and reverse nucleotide sequences of the primers for mediators are presented in Table 1.

### 2.8. Sensory Evaluation of Yogurt

To evaluate the acceptance of ginseng-supplemented probiotic yogurts, a sensory evaluation test was performed by 32 trained panelists aged from 21 to 40 years; the test was approved by the Institutional Review Board (approval number: IRB-SMU-C-4-004). Sensory characteristics, including color, texture, flavor, taste, and overall acceptance, were evaluated according to quantitative descriptive analysis [15]. The preference for each parameter was scored using a 7-point descriptive scale (1 = exceedingly unpreferable, 7 = exceedingly preferable).

### 2.9. Statistical Analysis

All experiments were performed in triplicate, and data are expressed as mean ± standard deviation. Statistical significance was analyzed with one-way analysis of variance (ANOVA) using SPSS software (version 18, SPSS Inc., Chicago, IL, USA). Duncan’s multiple range test was used to ascertain the degree of significant difference between samples or treatments at *p* < 0.05.

## 3. Results and Discussion

### 3.1. Ginsenoside Composition of Hydroponic Ginseng Extract

**Figure 1:**
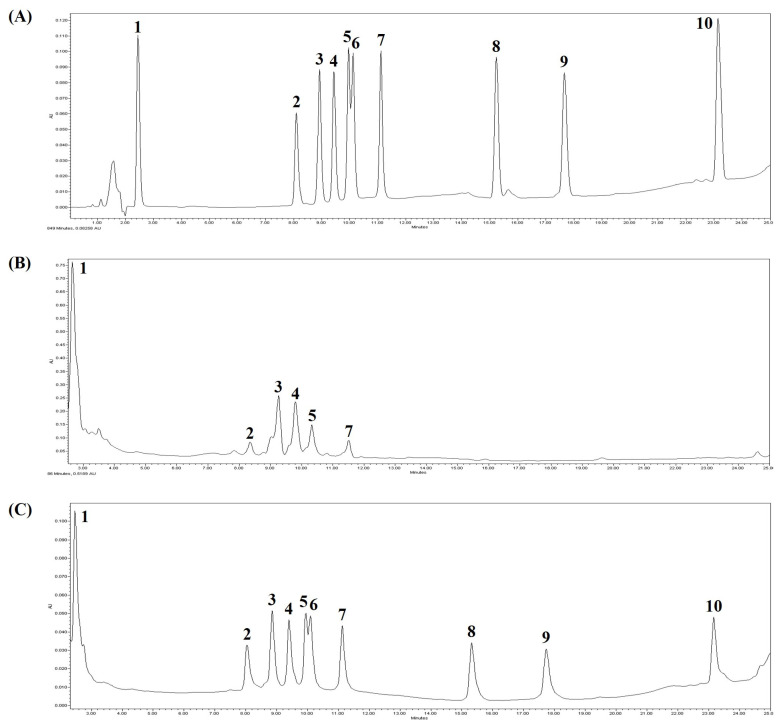
HPLC analysis results on ginsenoside composition of hydroponic ginseng extract. (**A**) Ginsenoside standards; (**B**) Hydroponic ginseng extract; (**C**) Mixtures of standards and hydroponic ginseng extract. 1, Re; 2, Rf; 3, Rb_1_; 4, Rc; 5, Rb_2_; 6, Rb_3_; 7, Rd; 8, F_2_; 9, Rg_3_; 10, CK. The *x*-axis represents retention time (0–25 min), and the *y*-axis represents AU (absorbance unit).

The ginsenoside composition of hydroponic ginseng extract was estimated by the HPLC analysis, and the results are shown in Figure 1. Ten kinds of ginsenoside standards were used to identify the ginsenoside composition of the samples. In addition, the mixture of standards and samples was also analyzed for accuracy verification. According to the result of Figure 1C, fortified ginseng extract represented the same tendency as the result of ginsenoside standard analysis without any other irrelevant peaks. After analysis, six kinds of ginsenoside, such as Re, Rf, Rb_1_, Rc, Rb_2_, and Rd, were detected, and their concentrations were 14.15, 3.17, 5.92, 5.24, 2.36, and 1.06 mg/g, respectively. The most abundant component within ginseng extract was ginsenoside Re, and the amount of ginsenoside Rb_1_ and Rc were second-highest compared to the rest of the ginsenosides. The chemical components of ginseng rely on species, geographical location, and climate. For example, *Panax ginseng* is mainly found in Korea and China, and, in the case of *Panax japonicus*, it is the dominant species of Japanese ginseng. Furthermore, *Panax quinquefolius* and *Panax trifolius* are mostly cultivated in North America. Moreover, *Panax notoginseng*, *Panax bipinnatifidus*, and *Panax stipuleanatus* are found across Southeast Asian countries [16]. Although they are the same species, the sampling region, extract method, total ginsenoside content, and ginsenoside components can be very diverse. For example, 10 batches of *Panax ginseng* grown in New Zealand were collected in pine forests around Taupo and Rotorua. However, the total content of ginsenosides ranged from 38.72 to 102.69 mg/g, and the major ginsenoside was also different from batch to batch [17].

In particular, ginsenoside Re, Rb_1_, Rb_2_, and Rd are known to have health-functional benefits in terms of antioxidant and anti-inflammatory activities [4]. Ginsenoside Re and Rf have the potential to be converted into minor ginsenoside Rh_1_, and ginsenosides Rb_1_, Rc, Rb_2_, and Rd can also be transformed into minor ginsenosides Rg_3_, F_2_, and CK. In our previous study, hydroponic ginseng extract was fermented by *L. brevis* B7 to transform the inherent ginsenoside into a more bioactive form. Although the fermented ginseng extract exhibited improved antioxidant and anti-inflammatory activities compared to non-fermented extract, changes in ginsenoside composition were not detected.

### 3.2. Changes in pH, Titratable Acidity, and Bacterial Growth during Yogurt Fermentation

**Figure 2:**
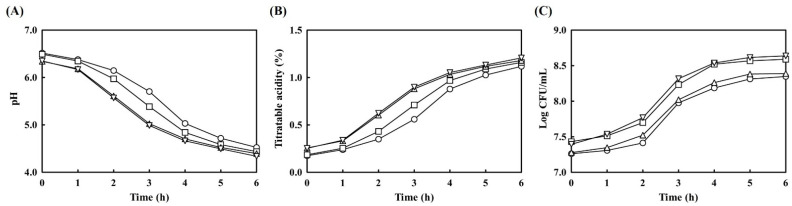
Changes in pH, titratable acidity, and bacterial growth in yogurt. (**A**) pH changes; (**B**) titratable acidity changes; (**C**) viable cell count changes. ◯, Non-additive-supplemented yogurt; □, *L. brevis* B7-supplemented yogurt; △, hydroponic ginseng extract-supplemented yogurt; ▽, *L. brevis* B7 and hydroponic ginseng extract-supplemented yogurt.

Changes in pH, titratable acidity, and total number of bacteria in the four types of prepared yogurts (control yogurt without any additives (C), *L. brevis* B7-supplemented yogurt (LB), hydroponic ginseng extract-supplemented yogurt (HG), and *L. brevis* B7 and hydroponic ginseng extract-supplemented yogurt (LHG)) were investigated during the fermentation period (Figure 2).

The initial pH was approximately 6.5 in C and LB yogurts, while a lower pH value of about 6.3 was observed in HG and LHG yogurts; these different results were likely caused by the slight sub-acidity of hydroponic ginseng (Figure 2A). Among ginseng components, several acidic polysaccharides may confer not only acidity but also immunomodulatory, antioxidant, and anti-fatigue activities [18]. The sub-acidity of ginseng affected the rapid decrease in pH; as a result, the fermentation of ginseng-supplemented yogurt was completed approximately 1 h prior to that of other yogurts. Additionally, the probiotic-supplemented LB yogurt showed a rapid decrease in pH compared to normal yogurt. This might be due to the growth of *L. brevis* B7, which can secrete several acidic compounds, such as lactic acid. The pH of yogurts supplemented with additives reached the value of 4.5 earlier than general yogurt, which took 6 h to complete fermentation.

The titratable acidity presented in Figure 2B showed an inversely proportional pattern compared to the pattern of changes in pH. The lowest titratable acidity was observed in C yogurt, while the highest result was observed in HG and LHG yogurts. These reverse correlations between pH and titratable acidity were also observed in a previous study [15].

The total number of bacteria is presented in Figure 2C. Owing to *L. brevis* B7 supplementation, which was identical to the inoculation amount of starter culture (approximately 10^7^ CFU/mL), the number of bacterial cells in LB and LHG yogurt was higher than that in other yogurts. Furthermore, it seemed that the growth of microorganisms might be influenced by HG extract supplementation as nourishment or prebiotics. However, the difference was less than 1.0 log CFU/mL; therefore, it was not possible to conclude that the HG extract significantly affected bacterial properties. However, it was thought that several ginseng compounds may have positive effects on microorganisms within yogurts.

### 3.3. Physicochemical Properties of Hydroponic Ginseng-Supplemented Probiotic Yogurts

The physicochemical and color properties of the yogurts were assessed; the results are presented in Table 2. The contents of fat, lactose, and ash did not show significant differences between the different types of yogurts. Although the protein contents of HG extract-supplemented yogurts (HG and LHG) were numerically increased, they were not significantly different from those in yogurts not supplemented with HG. The total solid content increased by approximately 0.55% in HG (15.52%) and LHG (15.43%) yogurts compared to that in C (14.93%) and LB (14.90%) yogurts. These increased solid contents could be attributed to the supplementation of HG extract. When compared to a previous study [15], The ranges of fat, protein, lactose, and total solid contents of control and hydroponic ginseng-added yogurts were 3.32–3.43%, 4.15–4.72%, 7.28–7.33%, and 14.71–15.42%, respectively. According to these results, it was considered that the prepared yogurt was consistently made, with similar quality. Furthermore, probiotics supplementation did not affect the other estimated parameters, which also implied that the *L. brevis* B7 strain did not influence the quality of yogurt.

Previously, similar studies were also performed to assess the possibility of ginseng-component-supplemented yogurts. To fortify the functional properties of yogurt, normal ginseng extract [19], red ginseng extract [20], and ginseng marc extract [21] were supplemented. The yogurt-supplemented non-processed ginseng [19] exhibited similar physicochemical properties (see Table 2). However, other cases showed different tendencies in protein and fat contents. The protein content decreased with the addition of ginseng marc extract [21], and the fat content decreased with the supplement of red ginseng extract [20]. Ginseng marc is the residue of ginseng dumped after the processing step, and red ginseng is processed ginseng produced by high-temperature treatment. Because of heat temperature and pressure, the structure of several ginsenosides changed and, thus, the diversity of ginsenosides also increased [22]. These different results could be attributed to the components of each extract. However, the decrease in protein and fat contents in ginseng yogurt compared to control yogurt was not clearly disclosed.

The results of color measurement are shown in Table 2. The color property was estimated with respect to three factors—L*, a*, and b*—which signify darkness to lightness, greenness to redness, and blueness to yellowness, respectively. A common trend in these results is that supplementation with *L. brevis* B7 does not show any influence on color properties; however, the HG extract supplement considerably affected all color factors. The lightness of HG (91.62) and LHG (91.74) yogurt was lower than that of C (93.61) yogurt because of the HG extract supplement. In contrast, both a* and b* values were increased by supplementation with the HG extract. The a* value increased, on average, by about 0.64 in HG and LHG yogurts; however, the difference was difficult to visualize with the naked eye. The b* value also increased roughly by 4.16 in HG and LHG yogurts compared with the C yogurt. Since these results were numerically and statistically meaningful, the difference could also be distinguished visually. Indeed, both the ginseng-supplemented HG and LHG yogurts were light brown in color, and non-ginseng-supplemented C and LB yogurts were light white in appearance.

### 3.4. Antioxidant Activities of Ginseng-Supplemented Probiotic Yogurts

**Figure 3:**
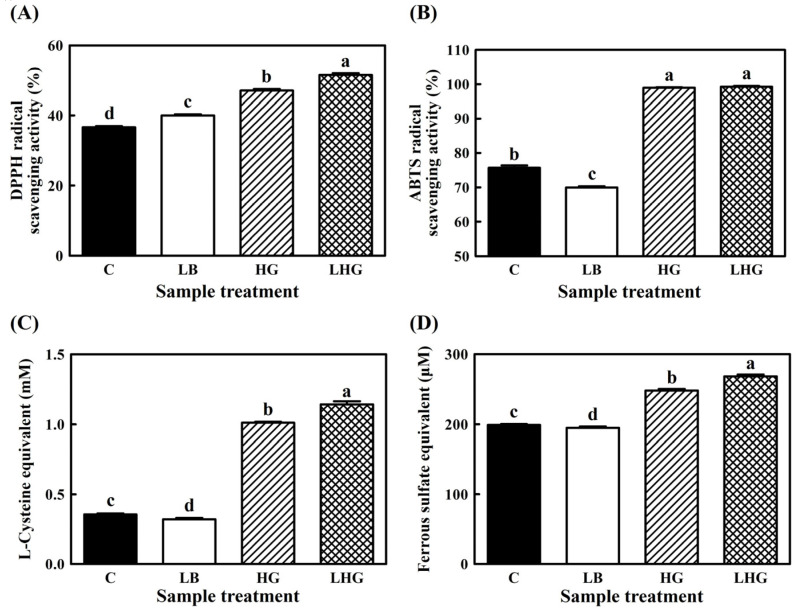
Antioxidant activities of hydroponic-ginseng-fortified probiotic yogurts. (**A**) DPPH radical-scavenging assay; (**B**) ABTS radical-scavenging assay; (**C**) reducing-power assay; (**D**) FRAP assay. ■, Control yogurt; □, *L. brevis* B7-supplemented yogurt; ▨, hydroponic ginseng extract-supplemented yogurt; ▩, *L. brevis* B7 and hydroponic ginseng extract-Scheme 0.

The antioxidant activities of the yogurts were estimated using the water-soluble extracts obtained via centrifugation and filtration. As shown in Figure 3, two kinds of radical-scavenging assays (DPPH and ABTS assays) and two kinds of ferric-reducing assays (reducing-power and FRAP assays) were performed.

A common feature of these results was that LHG yogurt consistently presented the highest antioxidant activities for all assays, and HG yogurt followed in second place. Both ginseng-supplemented HG and LHG yogurts showed significantly improved antioxidant activities compared to C yogurt. On the other hand, supplementation with *L. brevis* B7 did not improve antioxidant abilities, and the results were negative for this group except for those in the DPPH assay. These findings suggest that supplementation with *L. brevis* B7 has some adverse effects on the antioxidant functionality of yogurts. However, LHG yogurt containing both *L. brevis* B7 and hydroponic ginseng extract showed significantly improved antioxidant activity compared to non-probiotic HG yogurt (*p* < 0.05). This suggests that several chemical or microbial reactions occur during yogurt fermentation.

Ethanol-extracted ginseng has been reported to have hydroxyl and 2,2-diphenyl-1-picrylhydrazyl radical-scavenging and metal-ion-chelating activities [23]. Ginsenosides exhibit antioxidant activities in numerous ways. For example, ginsenoside Rg_3_ reduces excessive levels of ROS by suppressing the expression of HO-1/NQO-1, which is controlled by the Nrf-2 pathway [24]. In addition, ginsenosides Rd, Re, Rg_5_, and Rh_2_ inhibit the unnecessary expression of MDA and 4-hydroxynonenal, which are associated with lipid peroxidation [25].

Microbial fermentation can generate bioactive compounds, including antibiotics, phenolic compounds, and aromatic compounds. These compounds are regarded as secondary metabolites generated by plant fermentation and can be used in the food, cosmetics, and pharmaceutical industries [26]. Black beans are recognized as rich sources of isoflavones, carotenoids, anthocyanins, saponins, and vitamin E, with high antioxidant activities. These bioactive characteristics can be improved by fermentation using filamentous fungi, such as *Aspergillus* and *Rhizopus* species [27]. Mulberry is also a rich source of flavonoid compounds, such as quercetin and kaempferol, whose levels can be increased through fermentation by edible fungal species [28]. Furthermore, *Streptomyces rimosus* can generate anti-inflammatory oxytetracycline from groundnut shells [29], and *L. casei* can produce anti-fungal low-molecular-weight peptides from palm kernel cake [30].

Likewise, *L. brevis* B7, as well as three kinds of microorganisms within the starter culture, can act on ginseng components to produce functional compounds. Indeed, phenolic and flavonoid contents were increased after fermentation by *L. brevis* B7 (data not shown) and *L. mesenteroides* KCCM 12010P [3]. Therefore, the enhanced antioxidant activities of LHG yogurt compared to HG yogurt could be attributed to the elevated levels of the bioactive ingredients of *L. brevis* B7.

### 3.5. Cytotoxicity of Hydroponic Ginseng-Supplemented Probiotic Yogurts

**Figure 4:**
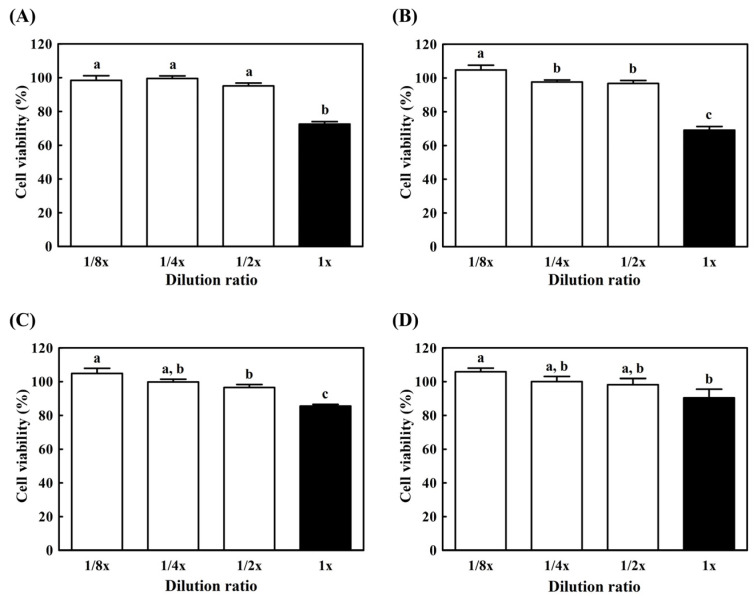
Cytotoxicity of hydroponic-ginseng-fortified probiotic yogurt on RAW 264.7 macrophage cells. (**A**) Control yogurt; (**B**) *L. brevis* B7-supplemented yogurt; (**C**) hydroponic ginseng extract-supplemented yogurt; (**D**) *L. brevis* B7 and hydroponic ginseng extract-supplemented yogurt. □, Cell viability higher than 95%; ■, cell viability lower than 95%. All data are presented as the mean ± standard deviation of values from triplicate experiments. Different letters above error bars indicate significant differences within the same assay (*p* < 0.05).

An MTT assay was performed to evaluate the cytotoxicity of supernatants of yogurts on RAW 264.7 cells before NO and RT-PCR analysis. The pH of each prepared water-soluble yogurt extract was adjusted to 7.0 and serially diluted from the initial concentration. As shown in Figure 4, the concentration of samples was diluted from 1 × to 1/8 ×; the clear-bar graph indicates cell viability higher than 95%. In the case of undiluted concentration, all types of yogurts presented weak cytotoxicity, with cell viability below 95%. On the contrary, a lower concentration corresponded to higher cell viability (>95%). Therefore, 1/2 × concentration was determined as the maximum condition for the following analysis.

Yogurt is considered a functional food with various health-improving activities, such as improving immune response, balancing gut microflora, preventing constipation and fungal/microbial infection, and anti-carcinogenic activity [31]. Numerous bioactive compounds are generated in milk during fermentation. Bioactive peptides can stimulate the proliferation and maturation of T lymphocytes and activate immune responses by elevating IgA levels [32]. Moreover, muramyl dipeptide promotes the secretion of anti- and pro-inflammatory cytokines through monocytes, lymphocytes, and macrophages [33]. Additionally, conjugated linoleic acid produced during yogurt fermentation plays an important role in immunomodulatory and anti-carcinogenic activities [34]. Likewise, these bioactive compounds produced during yogurt preparation exhibit strong functional properties and cytotoxic ability against cancer cells. Direct treatment of RAW 264.7 cells with yogurt samples that included bioactive compounds would be an irritant. Undiluted samples presented slightly stronger cytotoxicity in the results of the MTT assay. Based on these results, diluted yogurt samples were subjected to NO and immunomodulatory analyses.

### 3.6. Nitric Oxide Productivity of Hydroponic Ginseng-Supplemented Probiotic Yogurts

**Figure 5:**
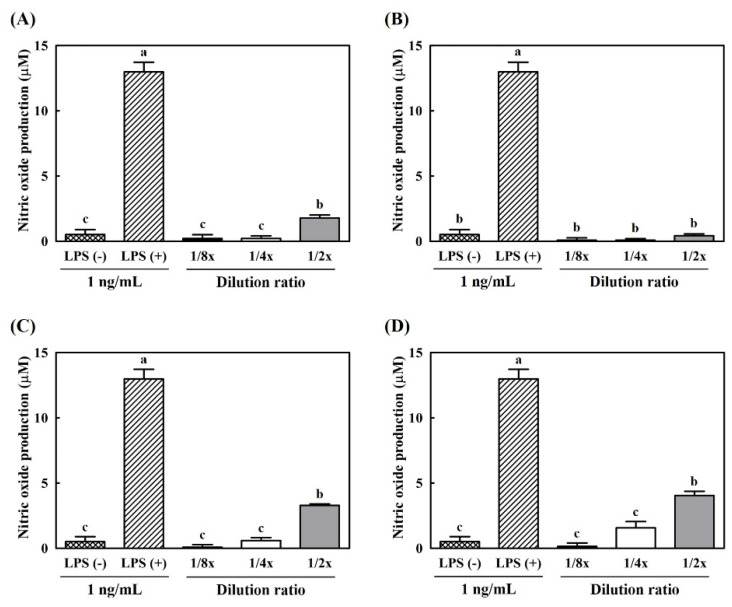
Nitric oxide inhibitory activity of hydroponic-ginseng-fortified probiotic yogurt on RAW 264.7 macrophage cells. (**A**) Control yogurt; (**B**) *L. brevis* B7-supplemented yogurt; (**C**) hydroponic ginseng extract-supplemented yogurt; (**D**) *L. brevis* B7 and hydroponic ginseng extract-supplemented yogurt. ▩, Without LPS treatment; ▨, with LPS treatment (1 ng/mL); ■, 1/8 × concentration; □, 1/4 × concentration; ■, 1/2 × concentration. Data are presented as the mean ± standard deviation of values from triplicate experiments. Different letters above the error bars indicate significant differences among samples (*p* < 0.05).

To evaluate the immunomodulatory potential of the yogurts, a NO assay was carried out. The NO assay results are presented in Figure 5; serially diluted yogurt supernatant samples at three different concentrations were tested. LPS-positive (1 ng/mL) and negative groups were designated as positive and negative controls, respectively. In addition, LPS was not added to the sample-treated group in order to evaluate the intrinsic immunomodulatory activity of the yogurt.

As shown in Figure 5, the amounts of secreted NO were 0.51 and 12.98 μM within LPS (−) and (+) groups, respectively. In the case of maximal concentration (1/2 ×) of the sample-treated group, NO was secreted at levels of 1.78, 0.43, 3.29, and 4.05 μM by the water-soluble extracts of C, LB, HG, and LHG yogurts, respectively (Figure 5A–D). However, NO was not secreted enough to induce immune reactions at lower concentrations, and the amounts of NO did not exhibit significant differences compared to those in the LPS-negative group. Therefore, the highest concentration (1/2 ×) was considered a suitable condition for subsequent real-time PCR analysis. In other studies, honey and rosella (*Hibiscus* species) with 4% concentration-supplemented yogurt exhibited immunomodulatory properties by producing 7.91 μM NO [35]; additionally, Bulgarian yogurts were reported to regulate immune response by the production of IFN-γ and the activation of NK cells [36].

### 3.7. Immunomodulatory Activities of Hydroponic-Ginseng-Supplemented Probiotic Yogurts

To investigate immunomodulatory activities, cytokines generated by RAW 264.7 macrophages were measured with real-time PCR analysis. Immunomodulatory factors, including TNF-α, iNOS, IL-1β, and IL-6, were measured, and β-actin was used as the housekeeping gene for RT-PCR analysis.

**Figure 6:**
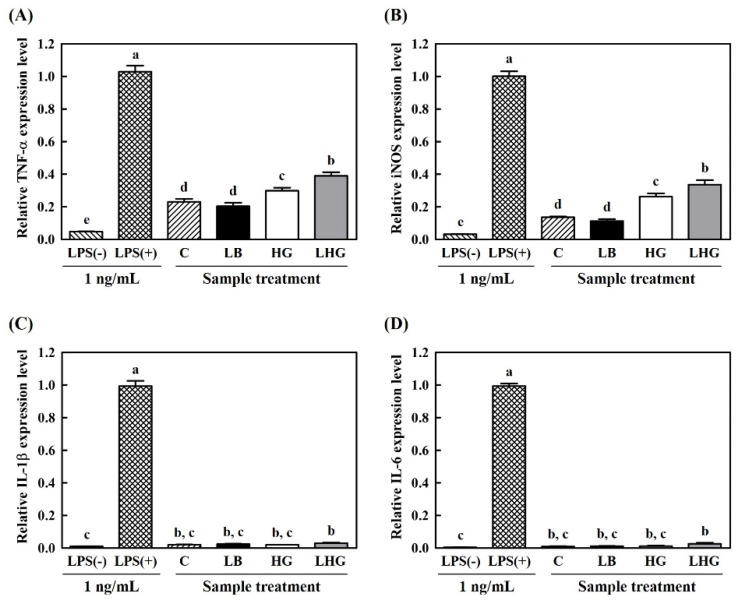
Immunomodulatory activities of hydroponic-ginseng-fortified probiotic yogurts in RAW 264.7 macrophage cells. (**A**) TNF-α; (**B**) iNOS; (**C**) IL-1β; (**D**) IL-6. ▧, Without LPS treatment; ▩, with LPS treatment (1 ng/mL); ▨, control yogurt; ■, *L. brevis* B7-supplemented yogurt; □, hydroponic ginseng extract-supplemented yogurt; ■, *L. brevis* B7 and hydroponic ginseng extract-supplemented yogurt. Data are presented as the mean ± standard deviation of values obtained from triplicate experiments. Different letters above the error bars represent significant differences among samples (*p* < 0.05).

The relative gene expression levels of the above-mentioned mediators, stimulated by the treatment with yogurt supernatants in RAW 264.7 cells, are presented in Figure 6. In terms of TNF-α and iNOS, gene expression levels were higher in LHG yogurt than in the C, LB, or HG yogurts. However, none of the yogurts induced the expression of IL-1β and IL-6. Although LHG yogurt stimulated a weak expression of IL-1β and IL-6 compared to the LPS-negative group, with significant differences (*p* < 0.05), no other significant differences were observed between the yogurt-treated groups.

These outcomes were correlated with the previous NO assay results. Higher amounts of NO were sequentially produced with the LHG, HG, C, and LB treatments. Furthermore, these sequential tendencies were observed in the results of TNF-α and iNOS expression. However, the amount of produced NO and the degree of gene expression were relatively lower than those reported in previous studies [6]. In these results, although the sample treatment stimulated the production of similar or higher amounts of NO as the LPS-positive group, the gene expression level in the sample-treated group was lower than that in the LPS-positive group. Likewise, yogurt treatments were insufficient to activate the genes of immune-related factors. These results may be attributed to the loss of solid fractions that were removed during the preparation of the water-soluble extract. Since various functional compounds are contained within the solid phase, a vast majority of immunomodulatory analyses can be carried out in vivo with intact forms of yogurts.

Nevertheless, the water-soluble extract of yogurt stimulated several immunological mediators, which could imply the presence of bioactive compounds. Milk and yogurt are considered the best suppliers of calcium, providing 40% of the daily nutritional requirement [37]. Milk fermentation can enhance the solubility of calcium, phosphorus, magnesium, and trace minerals. Solubilized calcium has been studied to improve the immunological activity of lymphocytes through lectin-binding ability and cytotoxic activity against tumor cells [38]. The anion exchange portion and dialysate isolated from yogurt are known to have anti-carcinogenic and anti-tumor activities through the activation of non-specific immune reactions [39]. *L. acidophilus* and *L. casei* have been reported to generate soluble compounds that activate immunity and prevent carcinogenesis and gastrointestinal disorders during milk fermentation [40]. Furthermore, the supernatant of yogurt, which is microorganism-free, has also been reported to activate NK cells and express IFN-γ in human peripheral blood lymphocytes [41].

In our study, yogurt fortified with *L. brevis* B7 and hydroponic ginseng exhibited increased antioxidant and immunomodulatory activities. In previous studies, co-fermentation of yogurt with fruits or vegetables has been attempted. Co-fermentation of carrot juice and starter culture consisting of lactic acid bacteria improved the viability of LAB and sensory characteristics with an orange color and delayed the decrease in pH during a 4-month storage period [42]. The extract of *Ligustrum robustum* Blume-fortified yogurt improved antioxidant activity and decreased the serum levels of malondialdehyde. Furthermore, it exhibited hypoglycemic activity via the amelioration of glucose tolerance in diabetic mice [43]. In another study, co-fermentation of the *Gnaphalium affine* extract in yogurts maintained high quality and enhanced radical-scavenging activity (superoxide, hydroxyl, DPPH, and ABTS radicals). Moreover, the activities of antioxidant enzymes, including catalases, glutathione peroxidase, and superoxide dismutase, were enhanced, and the level of malondialdehyde was substantially reduced [44].

### 3.8. Sensory Evaluation of Hydroponic-Ginseng-Supplemented Probiotic Yogurts

Sensory evaluation of hydroponic ginseng extract-supplemented probiotic yogurts was performed, and the results are shown in Table 3. The texture results did not show any statistical differences between the samples. Other factors were influenced by supplementation with the hydroponic ginseng extract. In this case, hydroponic ginseng supplementation was less appreciated compared to non-ginseng-supplemented C and LB yogurt. In a previous study, hydroponic ginseng was prepared under the same conditions, and sensory tests were carried out in a similar manner [15]. Even though the scores for HG yogurt presented in Table 3 are numerically similar to those in previous studies, the results were significantly different compared with those of the non-ginseng-supplemented yogurts.

Sensory evaluation is particularly subjective, and, thus, personal taste can be reflected in the results. Therefore, it was understood that differences in results could be influenced by individual variations. Supplementation with *L. brevis* B7 did not affect any evaluated factors when compared to non-probiotics-supplemented C and HG yogurts in the sensory test. The co-fermentation of hydroponic ginseng and probiotic *L. brevis* B7 during yogurt preparation did not positively influence the sensory evaluation, although the functional properties of the yogurt were significantly improved. It was considered that some negative opinions caused by private taste profiles would be tolerable in view of the health-enhancing aspects. Furthermore, these drawbacks could be improved by supplementation with sweetener treatments to reduce the distinct flavor of ginseng.

## 4. Conclusions

To date, several studies on the use of probiotics, ginseng, and fermented ginseng have been conducted; however, the application of probiotics and non-fermented ginseng together into yogurt, from the preparation stage, has been rare. In this study, the health-functional properties of yogurts prepared by co-fermentation with probiotic *L. brevis* B7 and hydroponic ginseng extract during yogurt preparation were investigated. The addition of both probiotics and ginseng influenced the rate of pH changes; in particular, the addition of hydroponic ginseng affected the total solid content, lightness, and yellowness of samples. Furthermore, the basic characteristics of yogurts were not significantly influenced by these additives. In the case of antioxidant assays, supplementation with hydroponic ginseng improved the antioxidant activities of yogurts, whereas *L. brevis* B7 supplementation did not generate any positive effects. However, the combination of *L. brevis* B7 and hydroponic ginseng showed the highest antioxidant activity. These results indicate that several bioactive compounds were generated by *L. brevis* B7 during yogurt preparation. Supplementation of both *L. brevis* B7 and hydroponic ginseng was not only ineffective to the cell viability of RAW 264.7 macrophages but also facilitative in the stimulation of nitric oxide. Furthermore, the hydroponic-ginseng-supplemented probiotic yogurt induced higher expression of immunomodulatory mediators, such as TNF-α and iNOS, than the other yogurts. Although ginseng-supplemented yogurts were scored lower on the sensory test, from a health-functional point of view, it was considered that it would be worthwhile to develop them as novel functional products, with convenience and accessibility in mind. Taken together, this study supports the feasibility of developing desirable functional food products with improved functional properties.

## Figures and Tables

**Table 1:** Primer sequences of immunomodulatory mediators and the reference gene for quantitative reverse transcription-PCR.

Primer ^1^	Acession Code	Direction	Primer Sequence (5′ to 3′)
β-Actin	NM_031144.3	Forward	GTGGGCCGCCCTAGGCACCAG
		Reverse	GGAGGAAGAGGATGCGGCAGT
TNF-α	XM_032888689.1	Forward	TTGACCTCAGCGCTGAGTTG
		Reverse	CCTGTAGCCCACGTCGTAGC
iNOS	AY211532.1	Forward	CCCTTCCGAAGTTTCTGGCAGC
		Reverse	GGCTGTCAGAGCCTCGTGGCTTTGG
IL-1β	NM_031512.2	Forward	CAGGATGAGGACATGAGCACC
		Reverse	CTCTGCAGACTCAAACTCCAC
IL-6	NM_012589.2	Forward	GTACTCCAGAAGACCAGAGG
		Reverse	TGCTGGTGACAACCACGGCC

^1^ TNF-α, tumor necrosis factor-α; iNOS, inducible nitric oxide synthase; IL-1β, interleukin-1β; IL-6, interleukin-6; β-Actin, reference gene.

**Table 2:** Physicochemical properties of hydroponic-ginseng-supplemented probiotic yogurts.

Sample Type ^1^	Nutritional Composition (%)					Color Parameter ^2^		
	Fat	Protein	Lactose	Total Solid	Ash	L *	a *	b *
C	3.27 ± 0.25	4.23 ± 0.25	7.57 ± 0.06	14.93 ± 0.16 ^b^	0.81 ± 0.03	93.61 ± 0.48 ^a^	−3.00 ± 0.11 ^b^	6.48 ± 0.20 ^b^
LB	3.20 ± 0.26	4.17 ± 0.21	7.53 ± 0.21	14.90 ± 0.18 ^b^	0.79 ± 0.06	93.93 ± 0.13 ^a^	−3.03 ± 0.04 ^b^	6.78 ± 0.10 ^b^
HG	3.27 ± 0.21	4.53 ± 0.32	7.57 ± 0.21	15.52 ± 0.33 ^a^	0.81 ± 0.04	91.62 ± 0.02 ^b^	−2.40 ± 0.09 ^a^	10.72 ± 0.04 ^a^
LHG	3.24 ± 0.35	4.47 ± 0.25	7.43 ± 0.12	15.43 ± 0.25 ^a^	0.82 ± 0.05	91.74 ± 0.01 ^b^	−2.33 ± 0.10 ^a^	10.55 ± 0.11 ^a^

^1^ C, control yogurt; LB, *L. brevis* B7-supplemented yogurt; HG, hydroponic ginseng extract-supplemented yogurt; LHG, *L. brevis* B7 and hydroponic ginseng extract-supplemented yogurt. ^2^ L *, lightness ranging from 0 (dark) to 100 (bright); a *, greenness to redness ranging from negative to positive values; b *, blueness to yellowness ranging from negative to positive values. ^a, b^ Different superscripts in the same factors imply significant differences (*p* < 0.05), analyzed with ANOVA. All data are presented as the mean ± standard deviation of values obtained from triplicate experiments.

**Table 3:** Sensory assessment results of hydroponic-ginseng-supplemented probiotic yogurts.

Evaluation Criteria	Sensory Evaluation of Each Type of Ginseng Yogurt ^1^			
	C	LB	HG	LHG
Color	6.10 ± 0.70 ^a^	6.13 ± 0.85 ^a^	4.97 ± 1.22 ^b^	5.03 ± 1.30 ^b^
Texture	5.97 ± 1.08	5.74 ± 1.03	5.61 ± 1.15	5.45 ± 1.09
Flavor	5.52 ± 0.96 ^a^	5.45 ± 1.12 ^ab^	4.90 ± 1.27 ^bc^	4.81 ± 1.05 ^c^
Taste	5.42 ± 1.03 ^a^	5.58 ± 1.12 ^a^	4.74 ± 1.26 ^b^	4.58 ± 1.48 ^b^
Overall acceptance	5.32 ± 0.60 ^a^	5.26 ± 0.89 ^ab^	4.81 ± 0.98 ^bc^	4.58 ± 1.06 ^c^

^1^ C, control yogurt; LB, *L. brevis* B7-supplemented yogurt; HG, hydroponic ginseng extract-supplemented yogurt; LHG, *L. brevis* B7 and hydroponic ginseng extract-supplemented yogurt. ^a–c^ Different superscripts within the same criteria indicate significant differences (*p* < 0.05), analyzed with ANOVA. All data are presented as mean ± standard deviation of values from triplicate experiments.

## Data Availability

Data is contained within the article.
